# Mothers employed in paid work and their predictors for home delivery in Pakistan

**DOI:** 10.1186/s12884-018-1945-4

**Published:** 2018-08-03

**Authors:** Sara Rizvi Jafree, Rubeena Zakar, Mudasir Mustafa, Florian Fischer

**Affiliations:** 1 Department of Sociology, Forman Christian College, Lahore, Pakistan; 2 Institute of Social and Cultural Studies, University of the Punjab, Lahore, Pakistan; 3 Department of Sociology, Institute of Social and Cultural Studies, University of the Punjab, Lahore, Pakistan; 4 Department of Public Health Medicine, School of Public Health, Bielefeld University, Bielefeld, Germany

**Keywords:** Mothers, Paid work, Employment, Delivery, Pakistan

## Abstract

**Background:**

Pakistan has one of the highest rates of maternal and neonatal mortality in the world. It is assumed that employed mothers in paid work will be more empowered to opt for safer institutional deliveries. There is a need to understand the predictors of home deliveries in order to plan policies to encourage institutional deliveries in the region.

**Methods:**

The study aimed to ascertain the predictors for home deliveries among mothers employed in paid work in Pakistan. Data analysis is based on secondary data taken from the Pakistan Demographic Health Survey 2012–13. Bivariate and multivariate logistic regression models were conducted.

**Results:**

The findings show that the majority (53.6%) of employed mothers in Pakistan give birth at home. Employed mothers in paid work with the following characteristics had higher chances for delivering at home: (i) women from rural areas (AOR 1.26; 95% CI: 0.94–1.71), or specific regions within Pakistan, (ii) those occupied in unskilled work (AOR 2.61; 95% CI: 1.76–3.88), (iii) women married to uneducated (AOR 1.70; 95% CI: 1.08–2.66), unemployed (AOR 1.69; 95% CI: 1.21–2.35), or unskilled men (AOR 2.02; 95% CI: 1.49–2.72), (iv) women with more than 7 children (AOR 1.57; 95% CI: 1.05–2.35), (v) women who are unable in the prenatal period to have an institutional check-up (AOR 4.84; 95% CI: 3.53–6.65), take assistance from a physician (AOR 3.98; 95% CI: 3.03–5.20), have a blood analysis (AOR 2.63; 95% CI: 1.95–3.57), urine analysis (AOR 2.48; 95% CI: 1.84–3.33) or taken iron tablets (AOR 2.64; 95% CI: 2.06–3.38), and (vi) are unable to make autonomous decisions with regard to spending their earnings (AOR 1.82; 95% CI: 1.27–2.59) and healthcare (AOR 1.12; 95% CI: 0.75–1.65).

**Conclusions:**

Greater efforts by the central and provincial state bodies are needed to encourage institutional deliveries and institutional access, quality and cost. Maternal and paternal benefits are needed for workers in both the formal and informal sectors of the economy. Finally, cultural change, through education, media and religious authorities, is necessary to support institutional deliveries and formal sector paid employment and out of home work opportunities for mothers of Pakistan.

## Background

Pakistan has one of the highest maternal mortality ratios [1] and neonatal mortality rates [2] in the world. Common causes of maternal mortality includes sepsis, severe anemia, obstetric hemorrhage, prolonged and obstructed labor, and hypertensive crises [3]. Intraventricular hemorrhage, hypothermia, encephalopathy, infant respiratory distress syndrome, breech presentation, injury to child during delivery, and preterm birth complications [4] are the common causes for neonatal mortality in Pakistan [4]. Most of these causes can be prevented through institutional deliveries [5, 6].

Employment of women is understood to be a pathway to autonomy and empowerment for women, especially in terms of gaining improvements in rights for decision-making and health for themselves and their children [7]. Research from low-income regions shows that uneducated, unskilled and rural women working in the informal sector of the economy predominantly opt for home deliveries [8, 9]. There are many socio-economic and cultural barriers preventing employed mothers in paid work (EMPW) from electing institutional deliveries including: high cost of healthcare, lack of permission from family, traditional norms, religious misinterpretation, and increased fear of caesarian deliveries [8, 10, 11].

Efforts by development bodies have targeted short term improvements to reduce maternal and child mortality by supporting women from rural and distant locations to give birth in their homes by skilled birth attendants or at local health centers with women attendants [12]. However, the problem with community facilitators delivering in homes or at small and under-resourced health centers is that mothers are unable to seek prenatal check-ups and consultancy from specialised medical practitioners (such as a sonographer, surgeon, nurse, physician, and lab experts) with complete institutional support. Institutional support includes x-ray and ultrasound machines, blood and urine tests, refrigerated medicine, uterotonic agents, scanning machines and surgical theaters for emergency caesarean sections, and sterile instruments [13, 14]. There is also the dilemma that despite the availability of trained midwives, women from conservative communities still opt for traditional birth attendants for assistance in home deliveries [15].

Pakistan has been unable to reduce maternal and under-five child mortality rates, unlike other countries in South Asia, and is lagging behind targets to meet the former Millennium Development Goals (MDGs) [16, 17] and the subsequent Sustainable Development Goals (SDGs). The report by National Institute of Populations Studies suggests that reasons for mortality rates remaining high in the country include the absence of prenatal trained medical practitioner consultation, and institutional check-up in monitoring the health of the mother and fetus [18]. Critical mortality risks that have been researched in the country include postpartum hemorrhage, retained placenta [19], sepsis and tetanus [20], diarrhea [21], preterm birth [22], asphyxia [23], and unhygienic delivery and infections [24]. It has been found that more than 95% of uterus ruptures occur in home deliveries [25], even though uterus ruptures are preventable in institutional deliveries. Similarly, neonatal tetanus is a common but avertable cause of death in Pakistani infants [5], and is caused when deliveries take place at home, using soil surface and also when there are sheep in the house [26].

In addition, home deliveries pose great health risks to the mother and child during the postnatal period, in terms of neglect of (i) colostrum provision and breast feeding practices [27], (ii) immunisations and nutrition supplementation for mother and child [28, 29], and (iii) overall postnatal care and institutional check-up of mother and child [30]. Previous research has shown that mothers, including employed and unemployed women, are more likely to give birth at home when they are uneducated, from poorer and rural backgrounds, when they have lower autonomy, and when the costs of institutional deliveries are high [31, 32].

Despite known risks, home deliveries prevail in conservative counties like Pakistan due to (i) trust and comfort with local birth attendant’s and community midwives [33], (ii) unfavorable services by inferior public hospitals, with distant locations, presence of male practitioners, lack of privacy and separation of child from mother after delivery [34], (iii) the belief that the traditional birth attendant (TBA) will handle the mother better and have her walking and back to work sooner than in institutional deliveries [35], (iv) and mistrust with medical practitioners and “western” or modern solutions for birthing, which is perceived to be a natural and ‘private’ process [36, 37].

International and local development bodies like the World Health Organization (WHO) and the National Maternal and Child Health Program have invested considerable resources in order to improve maternal and child health in Pakistan [21, 38]. They have been supported by Partners in Population and Development (PPD) and Partnership for Maternal, Newborn & Child Health (PMNCH) in encouraging disadvantaged women to gain employment through skill development and small business mobilisation [39]. Program objectives are to ensure progress toward achieving the SDGs in maternal and child health, most specifically in newborn mortality, infant mortality and maternal mortality. Presently, however, there is no mention specifically for targeted objectives to reduce neonatal mortality and measures to encourage institutional check-ups with trained medical practitioners during pregnancy to ensure safe deliveries.

To help rural women in unskilled and agriculture based employment to give birth safely, the government of Pakistan launched the National Maternal, Neonatal and Child Health program in 2006. The aim of the program was to train and place TBAs in rural areas; however, the deployment of these workers has been limited [40]. Also, both the local midwives and trained community health workers have been unable to significantly reduce maternal and child mortality [19]. In addition, many working and non-working mothers do not seek assistance from TBA, as they assume that their delivery will be normal [41]. There is still a need to understand the predictors for home deliveries for employed mothers in Pakistan in order to formulate adequate policies to support employed women in choosing institutional deliveries for their own and their child’s safety [42]. Up to now, there has been no study conducted exploring predictors for why employed mothers in Pakistan give birth at home. This research aims to identify: (i) the socio-demographic and pre-natal characteristics, and decision-making characteristics of employed mothers in paid work of Pakistan, and (ii) the association between employed mothers in paid work of Pakistan and home deliveries.

## Methods

### Data and sample

Secondary data from the Pakistan Demographic and Health Survey (PDHS) 2012–13, the third survey conducted in the country as part of the Measure DHS international series, has been used for this study. A total of 12,943 households were sampled and 13,558 ever-married women of reproductive age were sampled through multi-stage cluster sampling across Pakistan [18]. For this study, a total of 1387 EMPW with a child born in the last 5 years were included. Mothers with children born in the last 5 years were selected in order to avoid memory recall bias of the respondents.

### Variables included in analyses

The dependent variable was the place of delivery, distinguishing between home delivery and facility-based delivery.

The following socio-demographic characteristics were included in this study: age of employed mother, provincial belonging, regional belonging, education, wealth, marital status, occupation, husband’s education and occupation, total children born, head of household, and the place of delivery of last child born. Furthermore, seven independent variables were included in the analysis, which are based on a literature review: number of prenatal visits during pregnancy, prenatal assistance from physician, urine analysis taken during pregnancy, blood analysis taken during pregnancy, given/bought iron tablets during pregnancy, decision-maker for spending respondents’ money, and decision-maker for spending healthcare.

### Statistical analyses

Descriptive statistics were used to describe the socio-demographic characteristics of EMPW. Before including variables in multivariate analyses, we controlled for multicollinearity. The variance inflation factor for each independent variable covered a range between 1.04 and 3.69, indicating no multicollinearity among the variables included in the regression model. Bivariate logistic regression was conducted to show the predictors for home delivery by EMPW. The multivariate logistic regression model, was controlled for age, wealth and education of mothers. Odds ratios (OR) and Adjusted odds ratios (AOR) with 95% confidence intervals (CI) with a significance level of α = 0.05 were calculated.

## Results

### Socio-demographic characteristics

Table 1 shows the socio-demographic characteristic of EMPW in Pakistan, of which 45.4% were between 15 and 29 years, 45.3% were between 30 and 39 years and 9.2% were between 40 and 44 years. The mean age was 30 years and the standard deviation was 8.5 years. The majority of the employed mothers belonged to urban regions (65.8%), the uneducated strata (65.5%), and the poor wealth bracket (58.1%). A greater number of mothers (78.6%) were involved in unskilled or semi-skilled work (49.2 and 32.4%). Nearly all of the EMPW (98.3%) were currently married and living with their husbands. Around 96% women reported to live in a male headed household (who were their husband, father, or father-in-law). About half of the employed woman had between 1 and 3 children, and 53.6% delivered their children at home.

**Table 1:** Socio-demographic characteristics of employed mothers in paid work with children born in the last 5 years (*n* = 1387)

Characteristics	*n* ^*a*^	%
Age		
15–29 years	630	45.4
30–39 years	629	45.3
40–44 years	128	9.2
Province		
Baluchistan	195	14.1
KPK	136	9.8
Sindh	505	36.4
Punjab	551	39.7
Region		
Rural	475	34.2
Urban	912	65.8
Education		
No formal schooling	909	65.5
Primary	169	12.2
Secondary	124	8.9
Higher	185	13.3
Wealth		
Poor	806	58.1
Middle	226	16.3
Rich	355	25.6
Marital status		
Divorced/separated/widowed	23	1.7
Currently living with husband	1364	98.3
Mother’s occupation		
Unskilled	683	49.2
Semi-skilled	449	32.4
Skilled	254	18.3
Husband’s education		
No formal schooling	598	43.1
Primary	216	15.6
Secondary	340	24.5
Higher	232	16.7
Husband’s occupation		
Unemployed	24	1.7
Unskilled	798	57.5
Semi-skilled	333	24.0
Skilled	232	16.7
Total children born		
7 or more	270	19.5
4–6	474	34.2
1–3	643	46.4
Head of household		
Father/other	460	33.2
Husband	872	62.9
Self	55	4.0
Place of delivery		
Home	743	53.6
Hospital/clinic	644	46.4

^a^Some of the descriptive statistics do not add up to 1387 due to missing values in the data

Table 2 shows the descriptive statistics for prenatal health practices of EMPW. According to this, 71.7% of employed mothers had at least one prenatal visit during pregnancy to a health center, clinic, or hospital. However, only 37.6% sought assistance from a physician for prenatal check-ups. A significant number of EMPW had not taken samples for urine (48.4%) and blood analysis (52.2%) during pregnancy. In addition, almost 60% had not consumed iron tablets during pregnancy. Only half of employed mothers indicated that they could exclusively decide on how to spend their earnings, and very few (14.1%) indicated ability to make health decisions independently.

**Table 2:** Descriptive statistics for employed mothers in paid work and their reproductive health practices before delivery (*n* = 1387)

Characteristics	*n* ^*a*^	%
Number of prenatal visits during pregnancy		
None	393	28.3
At least one	994	71.7
Prenatal assistance from physician		
No	521	62.4
Yes	866	37.6
Urine analysis taken during pregnancy		
No	481	48.4
Yes	513	51.6
Blood analysis taken during pregnancy		
No	518	52.2
Yes	475	47.8
Given/bought iron tablets during pregnancy		
No	824	59.4
Yes	563	40.6
Decision-maker for spending respondent’s earning		
Husband or elders	227	16.3
Husbands, elders and self jointly	459	33.1
Self	701	50.5
Decision-maker for respondent’s healthcare		
‘Self and husband’ or ‘self and other’	1191	85.9
Self	196	14.1

^a^Some of the descriptive statistics do not add up to 1387 due to missing values in the data

### Simple bivariate logistic regression

Results of the bivariate logistic regression (Table 3) show that home deliveries were more likely to occur in Baluchistan (OR 4.79; 95% CI: 2.98–7.70), KPK (OR 3.07; 95% CI: 2.12–4.44), Sindh (OR 2.74; 95% CI: 1.89–3.94) as compared to Punjab. In addition, the likelihood for home deliveries was much higher in rural regions (OR 3.16; 95% CI: 2.50–3.98) than in urban areas. Employed mothers with no (OR 11.70; 95% CI: 7.56–18.10) or low (OR 2.45; 95% CI: 1.76–3.42) education were also significantly more likely to give birth to their children at home. Mothers from poor (OR 6.56; 95% CI: 4.94–8.72) and middle income (OR 2.17; 95% CI: 1.61–2.93) also had a higher likelihood for home deliveries than women from highest wealth group.

**Table 3:** Simple bivariate logistic regression for predictors of home delivery among mothers employed in paid work

Characteristics	OR (95% CI)	*p*-value
Age		
15–29 years	1.33 (0.90–1.96)	0.151
30–39 years	1.14 (0.48–2.69)	0.578
40–44 years	1	
Province		
Baluchistan	4.79 (2.98–7.70)	< 0.001
KPK	3.07 (2.12–4.44)	< 0.001
Sindh	2.74 (1.89–3.94)	< 0.001
Punjab	1	
Region		
Rural	3.16 (2.50–3.98)	< 0.001
Urban	1	
Education		
No formal schooling	11.70 (7.56–18.10)	< 0.001
Primary	3.24 (2.19–4.78)	< 0.001
Secondary	2.45 (1.76–3.42)	< 0.001
Higher	1	
Wealth		
Poor	6.56 (4.94–8.72)	< 0.001
Middle	2.17 (1.61–2.93)	< 0.001
Rich	1	
Marital status		
Divorced/separated/widowed	1.51 (0.66–3.46)	0.330
Married	1	
Mother’s occupation		
Unskilled	6.51 (4.67–9.07)	< 0.001
Semi-skilled	1.86 (1.45–2.37)	< 0.001
Skilled	1	
Husband’s education		
No formal schooling	6.60 (4.66–9.36)	< 0.001
Primary	2.15 (1.63–2.82)	< 0.001
Secondary	1.43 (1.04–1.97)	0.028
Higher	1	
Husband’s occupation		
Unemployed	4.79 (1.93–6.89)	0.001
Unskilled	3.56 (1.42–8.93)	0.007
Semi-skilled	1.29 (0.53–3.17)	0.566
Skilled	1	
Total children born		
7 or more	2.32 (1.73–3.12)	< 0.001
4–6	1.12 (0.82–1.52)	0.468
1–3	1	
Head of household		
Father/Other	0.94 (0.54–1.62)	0.824
Husband	1.26 (0.72–2.21)	0.413
Self	1	
Number of prenatal visits during pregnancy		
None	7.72 (5.71–10.43)	< 0.001
At least one	1	
Prenatal assistance from physician		
No	6.10 (4.75–7.85)	< 0.001
Yes	1	
Urine analysis taken during pregnancy		
No	3.98 (3.04–5.21)	< 0.001
Yes	1	
Blood analysis taken during pregnancy		
No	4.30 (3.27–5.66)	< 0.001
Yes	1	
Given/bought iron tablets during pregnancy		
No	4.11 (3.27–5.16)	< 0.001
Yes	1	
Decision-maker for spending respondent’s earning		
Husband or elders	2.47 (1.77–3.44)	< 0.001
Husband, elder and self jointly	2.36 (1.66–3.33)	< 0.001
Self	1	
Decision-maker for respondent’s healthcare		
Self and husband’ or ‘self and other’	1.87 (1.32–2.65)	< 0.001
Self	1	

Employed mothers in unskilled work (OR 6.51; 95% CI: 4.67–9.07) or semi-skilled work (OR 1.86; 95% CI: 1.45–2.37) showed higher odds of delivering at home. Similarly, mothers with husbands who were unemployed (OR 4.79; 95% CI: 1.93–6.89) or employed in unskilled work (OR 3.56; 95% CI: 1.42–8.93) also had higher odds of delivering at home. EMPW with husbands who had no formal schooling (OR 6.60; 95% CI: 4.66–9.36), or with primary (OR 2.15; 95% CI: 1.63–2.82) and secondary (OR 1.43; 95% CI: 1.04–1.97) schooling had higher odds of home deliveries. Mothers with 7 or more children (OR 2.32; 95% CI: 1.73–3.12) or 4–6 children (OR 1.12; 95% CI: 0.82–1.52) have higher likelihood of delivering at home.

Mothers without institutional prenatal check-ups (OR 7.72; 95% CI: 5.71–10.43) and those who had not taken prenatal assistance from a physician (OR 6.10; 95% CI: 4.75–7.85) were more likely for delivering at home. Employed mothers who had not had urine or a blood analysis and did not consume iron supplements during pregnancy showed a higher likelihood to give birth to their children at home. Lastly, EMPW with decision-making for spending their earnings (OR 2.47; 95% CI: 1.77–3.44) and healthcare (OR 1.87; 95% CI: 1.32–2.65) being controlled by others (husbands or elders) or jointly (‘self and husband’ or ‘self and other’) had higher odds of home delivery.

### Multivariate logistic regression

In the multivariate logistic regression adjusting for mother’s age, education, and wealth status, most of the socio-demographic variables were identified as covariates and thus showed no significance (Table 4). However, the model confirms the relevance of reproductive health practices associated with the place of delivery. According to these results, employed mothers who (i) had not been through a prenatal check-up (AOR 4.84; 95% CI: 3.53–6.65), (ii) had not taken prenatal consultancy from a physician (AOR 3.98; 95% CI: 3.03–5.20), (iii) did not have urine analysis (AOR 2.48; 95% CI: 1.84–3.33), (iv) did not have blood analysis (AOR 2.63; 95% CI: 1.95–3.57) or (v) had not taken iron supplements (AOR 2.64; 95% CI: 2.06–3.38) during pregnancy were more likely to give birth at home. Finally, EMPW who were unable to make autonomous decisions related to (i) spending their earnings [husbands or elders made the decision (AOR 1.82; 95% CI: 1.27–2.59); husbands, elders and self jointly made the decision (AOR 1.88; 95% CI: 1.29–2.73)] and, (ii) spending on healthcare (AOR 1.12; 95% CI: 0.75–1.65), have higher odds of giving birth at home.

**Table 4:** Multivariate logistic regression for predictors of home delivery among mothers employed in paid work

Characteristic	AOR^a^ (95% CI)	*p*-value
Province		
Baluchistan	1.63 (1.42–1.88)	< 0.001
KPK	1.73 (1.46–2.05)	< 0.001
Sindh	0.87 (0.78–0.98)	0.021
Punjab	1	
Region		
Rural	1.26 (0.94–1.71)	0.122
Urban	1	
Marital status		
Divorced/separated/widowed	0.62 (0.25–1.49)	0.282
Married	1	
Mother’s occupation		
Unskilled	2.61 (1.76–3.88)	< 0.001
Semi-skilled	1.23 (0.94–1.61)	0.121
Skilled	1	
Husband’s education		
No formal schooling	1.70 (1.08–2.66)	0.020
Primary	1.16 (0.85–1.58)	0.335
Secondary	1.10 (0.79–1.54)	0.551
Higher	1	
Husband’s occupation		
Unemployed	1.69 (1.21–2.35)	0.002
Unskilled	2.02 (1.49–2.72)	< 0.001
Semi-skilled	0.52 (0.19–1.37)	0.518
Skilled	1	
Total children born		
7 or more	1.57 (1.05–2.35)	0.026
4–6	0.92 (0.65–1.32)	0.682
1–3	1	
Head of household		
Father/other	1.07 (0.58–1.97)	0.816
Husband	1.00 (0.77–1.30)	0.978
Self	1	
Number of prenatal visits during pregnancy		
None	4.84 (3.53–6.65)	< 0.001
At least one	1	
Prenatal assistance from physician		
No	3.98 (3.03–5.20)	< 0.001
Yes	1	
Urine analysis taken during pregnancy		
No	2.48 (1.84–3.33)	< 0.001
Yes	1	
Blood analysis taken during pregnancy		
No	2.63 (1.95–3.57)	< 0.001
Yes	1	
Given/bought iron tablets during pregnancy		
No	2.64 (2.06–3.38)	< 0.001
Yes	1	
Decision-maker for spending respondent’s earning		
Husband or elders	1.82 (1.27–2.59)	0.001
Husbands, elders and self jointly	1.88 (1.29–2.73)	0.001
Self	1	
Decision-maker for respondent’s healthcare		
‘Self and husband’ or ‘self and other’	1.12 (0.75–1.65)	0.058
Self	1	

^a^Multivariate logistic regression analysis was carried out to obtain AOR after controlling for mother’s age (continuous variable), education (categorical variable) and wealth status (categorical variable)

## Discussion

The findings show that the majority of women employed in paid work in Pakistan give birth to their children at home, placing themselves and their child at risk of mortality. Research from high-income countries has shown that institutional deliveries reduce neonatal mortality, due to the presence of medical technologies [43]. We found that employed mother in rural areas and the provinces of KPK and Baluchistan are more likely to give birth at home, due to fewer rural and provincial structural healthcare support and policies, and possibly the sustenance of inflexible traditional practices [31]. Results indicate that provincial political devolution can have its limitations, in that maternal and child health programs are not having symmetrical improvements across provinces [44]. It was also found that EMPW who are employed in unskilled and semi-skilled work are more likely to give birth to their children at home. The implication is that most of the employed mothers in Pakistan are not gaining from the benefits of formal sector employment, with maternal benefits; which might be a strong contributing factor for lack of prenatal check-ups and institutional deliveries [45]. Our findings corroborate other literature from low-income countries, like Bangladesh, Kenya and Ghana, in that employed mothers in unskilled work are (i) unaware of the benefits of institutional delivery, (ii) unable to access institutional and trained healthcare practitioner assistance, and (iii) involved in the informal sector of the economy that provides them fewer health benefits, such as health insurance or medical allowances [10, 46, 47].

A significant predictor of home delivery found in our study was the absence of prenatal check-ups and consultation from physicians. Other research confirms that employed mothers with lack of prenatal consultancy and trained healthcare practitioner visitation are less likely to opt for institutional delivery, due to lack of guidance and skilled medical monitoring [48]. The main benefit of prenatal check-ups with physicians includes plans for safe delivery and timely referral for institutional delivery if risks are present. Research from neighboring India highlights that taking prenatal assistance from non-physicians can place the mother and child at more risks of not opting for institutional deliveries and mortality hazards from physical, environment, and cultural factors [49]. Additionally, trained care providers and LHWs attempting to provide healthcare for mothers and children complain of inability to deliver services in the community due to family resistance and obstruction, which is a patient safety hazard [50].

Our results support findings from India that employed mothers who, during pregnancy, did not have urine or blood analysis, or receive iron supplements were more likely to give birth to children at home [51, 52]. These findings imply that EMPW need prenatal services from experienced and skilled health providers in order to secure institutional deliveries and reproductive health. It is also empirically evidenced that when mothers are able to secure health through institutional consultation during the prenatal period they are more likely to opt for post-natal check-ups and nutritional supplementation, newborn check-ups and immunisation, and early breastfeeding practices [53].

We also found that mothers with seven or more children are more likely to give birth at home. Other literature from the region suggests that women are less likely to give birth in institutions after the birth of their first child [54]. This may be because of cost issues, lack of time to pursue prenatal check-ups, and the belief that multiple births make the mother, and community midwives, experienced enough to manage home deliveries [55]. In fact, mothers with multiple children are at risk of self and child mortality during home delivery due to complications caused by repeat pregnancies and physical weakness of the mother [56].

Our results highlight that the literacy and employment of husbands plays a key role in the health of the mother and child. Other literature from low-income countries, like Nepal and Nigeria, confirms that more educated and skilled husbands with higher pay (i) are more aware of the health risks of home deliveries, (ii) are more able to resist regressive traditional practices of the family and kin, and (iii) have more money to support institutional delivery costs [57, 58].

Of utmost concern is the finding that EMPW in Pakistan are unable to autonomously make decisions with regard to spending their earnings, and are also unable to make independent decisions regarding their health practices. Studies, from Nepal and Sialkot, show that many employed mothers are unable to autonomously control spending on health and household purchases [59, 60]. Scholars and experts agree that when cultural constraints prevent employed women from making their own decisions, their financial autonomy is of benefit primarily to their husbands, in-laws and male relatives, and not their self and children [61]. This can result in multiple challenges for them extending beyond reproductive health, such as inability to control: large and small household consumption, education of their children, early marriage of their daughters, domestic and intimate partner violence, and overall psychological stability of self and child.

The main limitations of this study are associated with the usage of secondary data, which are e.g. the inability to investigate other variables that may influence home deliveries, such as permission from in-laws, traditional customs and the social stigma of institutional deliveries. Also, this study has not been able to investigate the problems unemployed mothers or employed mothers without paid cash, especially in the rural regions, may face in preventing institutional deliveries. In addition, the data rely on a cross-sectional study design, which do not allow for causal inferences. However, this study has its strengths. Significant predictors of home deliveries have been found which will help policy makers to develop improved programs to meet the SDGs.

## Conclusions

Pakistan needs more long-term solutions for encouraging institutional deliveries. Public health strategies involving encouragement of institutional consultation during the prenatal period might be the most important vehicle for reducing maternal and child mortality rates in low-income and conservative societies [15]. Central state and provincial bodies need to promote institutional deliveries across provinces and rural areas in terms of access, cost, subsidisation, and quality. Assistance in developing cash payments and employer coverage of institutional deliveries for both working mothers and fathers is needed. Finally, increase in institutional deliveries and health decision-making of mothers requires a change in the cultural mindset of the nation, with combined structural and civilian mobilisation across sectors like education and media, and agencies like community notables and religious authorities [61].

## Abbreviations

AOR: Adjusted odds ratio
CI: Confidence interval
EMPW: Employed mothers in paid work
MDG: Millennium Development Goal
MNCH: Maternal, newborn and child health
OR: Odds ratio
PMNCH: Partnership for Maternal, Newborn & Child Health
PPD: Partners in Population and Development
SDG: Sustainable Development Goal
TBA: Traditional birth attendant
WHO: World Health Organization
